# 

**Published:** 1891-08-29

**Authors:** 


### NOTICE TO CORRESPONDENTS.

All MS., Letters, Books for Review, and other matters intended for the Editor should be addressed The Editor, The Lodge, Porchester Square, London, W.

The Editor cannot undertake to return rejected M.S., even when accompanied by stamped directed envelope.

**Notice to Advertisers and Subscribers.**

All Advertisements, Orders for Copies of The Hospital, and Business Communications should be addressed to The Publisher, *not to the Editor*, The Hospital, 140, Strand, W.C.

The Cost of Subscription, post free, is as follows:—Per week, 1½d.; per quarter, 1s. 8d.; per half-year, 3s. 3d.; per annum, 6s. 6d. Foreign:—Per annum, 8s. 8d.; payable, in all cases, in advance.

**NOTICE.—The Index for Vol. IX.. (ending March, 1891) is on sale at the Office of this Journal, price 2½d., post free.**

### General Charities.

[This Column is devoted to an account of work of charitable institutions other than Medical Charities. The Editor will be glad to receive reports or news of work at 140, Strand. *Philanthropy* should be plainly written on the envelope.]

The following, among other institutions, have benefited under the will of the late Mr. Henry A. Brassey: The Infant Orphan Asylum at Wanstead, the Royal Albert Orphan Asylum, Bagshot, the Academy for the Blind, and the Orphan Working School, Haverstock Hill. Each receive a legacy of £500. Mr. Brassey has also bequeathed the sum of £500 to the National Refuges for Homeless and Destitute Children, Great Queen Street, to be primarily applied in aid of maintaining the boys in and upon, and in keeping up the training-ships "Chichester" and "Arethusa."

Not the least known among industrial institutions is the **Training Ship "Cornwall,"** which was certified 31 years ago for 200 inmates. It is creditable to the management, that out of 285 boys sent into the world during three years only seven have been reconvicted, and that so many old inmates revisit the ship. During the past year the average number of boys maintained has been 214, and the industrial profits have amounted to £212 18s. 3d. The training the boys receive is a thoroughly practical one, including carpentering, tailoring, shoemaking, and sail-making. Naturally a great number go to sea, but many join various trades on land, and few, curiously, as we pointed out in writing of the "Arethusa" join the Royal Navy. Donations will be gratefully acknowledged by the Captain Superintendent, the "Cornwall," off Purfleet.

The education and training of children under the difficulties of extreme poverty, and of bad parents and vicious surroundings, have presented many hard problems for solution. Those who have reflected on the enormous sums paid by the country in support of gaols and reformatories have felt how the necessity for these institutions has arisen, to a great extent, owing to the neglect of children who are allowed to grow up without either attention or care. With a view to meet the necessity of a moral and industrial training for those children who have been born into the world unblessed with parents able to fulfil their duties in consequence either of extreme poverty or of their profligate lives and evil surroundings, industrial schools were established. It was pointed out forcibly some few years ago by Mr. Samuel Smith, M.P., that "millions of people exist in our midst unable to live except upon charity, because they have been taught in youth no means of livelihood. The little smattering of knowledge got in our schools by the children of this class is rubbed off them in the critical years that succeed school-life; it only enables them to read the *Police News*, the *Newgate Calendar*, and such-like rubbish." The Board schools have not done all that was hoped for them, but they have just given sufficient education to their pupils to enable them to read and enjoy cheap and sensational trash without giving them the power to distinguish between what is healthy and bad. The Ragged schools, on the other hand, have shown that it is possible to educate and humanise even the lowest and most wretched children, and much valuable work is now being done in this direction by the Industrial schools, of the principal of which we propose to give a slight sketch. They give industrial capacity for independence, and, if properly organised, do not throw their pupils on the sea of life till they are thorough workmen. A difficulty which, for many years, industrial school managers have had to contend with, and which has been recognised by the Government Inspectors, is the injury sustained by scholars from the interference of bad parents, who, though quite willing and thankful to accept the help of industrial schools during the unprofitable period of their children's lives, take the children away from the school the moment they are able to earn money, in order to place them in some employment, irrespective of any prospect of advancement. It is, to be hoped, however, that the Bill recently passed into Law, will remedy some of the evils which exist, even though it may not compel parents whose neglect has brought their children to industrial schools to pay at least some part of the cost while their children were being trained to an industrial life. In addition to the 141 industrial schools now under inspection there now exists another agency for the reclamation of children in the form of truant schools. These schools are intended for children, who, after due warning, have failed to make a satisfactory number of attendances at the ordinary day schools. As pointed out by H.M. Inspector Lieut.-Colonel Inglis, they do good in two ways—exercising on the boys committed to them a most wholesome influence, enforcing habits of punctuality, regularity, and obedience, probably for the first time in their lives. At the present time there is a great want of uniformity in the system of management applied to these schools. In some a boy has to undergo a short term of solitary confinement on his first visit, and a more lengthened one on each recommitment. In other schools this system has never been adopted, and rightly. The practice of solitary confinement is a pernicious and cruel one, which has long been acknowledged as bad for adults, and must, therefore, be infinitely worse for a child, and should be abolished in every school. The results of such a system can work no possible good, but they can and do lead to great misery and evil. We had thought that this antiquated system of severity was as extinct as the dodo, but it is a fact that in some of these schools it is carried so far that no play is ever allowed, drill being substituted in its place!

### Scraps and Gleanings.

The Marlborough Hospital Sunday Fund have handed over their collection of £11 to the Savernake Hospital.

The South Coast Medical, Surgical, and Convalescent Home is about to be moved to larger and brighter quarters.

A trained nurse has been secured to work amongst the sick poor of Tenby. Money to defray the expenses of her services has been subscribed in the neighbourhood.

At the annual meeting of the Burton Cottage Hospital a very satisfactory report was presented. The institution has proved a great boon to the neighbourhood.

Ulverston Cottage Hospital presents a satisfactory report for the past year, and shows a balance of £135 on its accounts. Much interest in the hospital is shown in the neighbourhood.

The annual meeting of subscribers to the Halstead Cottage Hospital was held at the Town Hall on Tuesday afternoon. The report showed the hospital to be in a very flourishing condition.

The disgraceful scenes witnessed at the Hospital Sunday demonstration at Wantage have caused great offence.

The twentieth annual report of the Horton Infirmary, Banbury, testifies to the continued popularity of the institution.

The eighteenth annual report of the Ingham Infirmary, South Shields, records that the funds of the institution are in a highly satisfactory condition, the balance-sheet showing a balance on the year's working of £93 18s. 2d., as against £98 9s. 7d. in the previous year.

"Hospital Saturday," a movement so recently started in Dover, has sprung into popular favour, and this year has seen its rapid development and a consequent increased collection.

It takes a good many "littles" to make a "mickle"—sometimes. The contents of one box used in collecting for the Brighton Hospital Sunday Fund weighed 36 pounds. It contained the sum of £4 6s. We should imagine the box was especially devoted to receiving the widow's "mite."

The report of the National Training School of Cookery, Buckingham Palace Road, has just been issued. It appears from it that since the opening of the school the total number of pupils has amounted to 40,407.

To what pitch of fancy will advertisers resort to next? The *Hospital Gazette* publishes an advertisement for the Liquor Carnis, a parody on Gilbert's lines in the "Gondoliers," the refrain of which is "No possible doubt whatever." This improved edition is entitled, "The true story of the Gondoliers."

The Bridlington Sloyd Cottage Hospital has been very fortunate in kind friends during the past year. £90 has been received as a legacy, £100 donation from Miss Lloyd, of Stockton Hall, and £20 from the Bridlington Charity Trustees, besides large sums, the proceeds of entertainments on behalf of the hospital.

Vegetarians must be shocked at the behaviour of the Kea parrot. It seems that this abandoned fowl, who lives in New Zealand, was accustomed to feed upon fruit. But since the introduction of sheep the Kea parrot has gone back on his principles, and now lives exclusively on mutton, and chiefly on mutton kidneys.

The fourth annual Hospital Sunday demonstration in aid of the Hertford Infirmary, the Herts Convalescent Home, and the Hertford and Bengeo Nurses' Fund took place at Hertford on the 9th ult. The financial results, so far as they are known at present, are satisfactory, the total raised being £106 6s. 7d., but other amounts have still to come in.

A man living at Portslade has just accomplished a novel feat. He made a wager to eat eight pounds of potatoes under an hour, and he performed his task at the Gardener's Arms, Portslade, in the space of twenty-seven minutes. Notwithstanding his meal he expressed himself ready to eat another four pounds, and afterwards attended a smoking concert.

The Mayor of Portsmouth (Sir William Pink), presiding at the sixth annual meeting of the Portsmouth and South Hants Eye and Ear Infirmary, observed that the Infirmary had been instrumental in effecting a great work in the Borough. From the date of its establishment up to the present time the patients have averaged between 1,800 and 1,900 a year—about 11,000 in all.

The annual collections organised by the Bournemouth Borough and Friendly Societies Hospital Saturday and Sunday Committee took place on Saturday and Sunday, and were attended by a considerable amount of success. The workshop returns have not yet been sent in, but the street collection realised £17 4s., a slight increase on the previous year, and the whole collection amounted to over £90.

A "flower-match" formed an interesting item the other day in connection with a school-treat on the Sussex Downs. Twenty-eight girls took part in the competition. They were allowed two hours to scamper over the hills in search of botanical specimens. The first prize was awarded to a child bearing the happily appropriate name of Lily Rose, who presented her judges with fifty-five specimens.

The annual Church Parade of the Aylesbury United Benefit Societies, on behalf of the Bucks Infirmary, took place on Sunday, the 9th ult. Unhappily the weather was very inclement, rain falling continually during the greater part of the day, and all the time the parade was taking place. £24 was collected, and after deducting the expenses, amounting to £4, a balance of £20 remained, which it was decided to hand over to the Infirmary.

The workmen of Messrs. Ropner and Son's ship-yard have generously given a donation of nearly £20, in addition to having agreed to subscribe 1d. per month per man, to the Stockton-on-Tees Nursing Association, and the same systematic subscription has been begun at the West Stockton Ironworks. The workmen employed in other manufacturing works of the district, who have already given donations, are also favourably considering the proposal to subscribe regularly.

The North Devon Infirmary has had an unfortunate financial year. The income of the institution has been exceeded by £159 17s. 7d., whilst the receipts have this year been less than they have been for many years past, the decrease being £215 4s. 3d. No legacies have been received, and the donations were much smaller. The expenditure had been much increased by a heavy bill for doing up the interior of the Institution. The building was now in a highly satisfactory state.

### The Student of Medicine.

[All communications for this Supplement should be addressed to The Editor of The Hospital, at 140, Strand, with the words, "Students' Column," written in the left hand top corner of the envelope.]

### SYPHILIS: ITS TRANSMISSION AND TREATMENT.

By C. Gordon Brodie, F.R.C.S., Assistant Surgeon to the North-West London Hospital; Senior Demonstrator of Anatomy, Middlesex Hospital.

*Abstract of a paper read before the Middlesex Hospital Medical Society.*

In these brief notes, which have been culled from various sources, I shall endeavour to deal with some of the sources of infection of syphilis, and to show how a very small act of carelessness may produce a spread of this fell disease, as well as to describe some of the rare ways in which the disease has been transmitted, of which a few authentic cases are on record. It must be borne in mind, however, that in the greater majority of cases where the patient wishes to conceal the true method of infection, all sorts of excuses are made to try and mislead the unwary. Although, as a rule, the motto *honi soit que mal y pense* may often be applied in surgery and medicine; yet where facts point one way and the history another, we must stand by the former and allow the latter to go to the wall. The best definition of syphilis is that of Mr. Hutchinson, viz., "a specific disease or fever of prolonged but definite stages which is produced by contagion only, which has a period of incubation, a period of outbreak (known as the primary symptoms), a period of exanthem or efflorescence (known as the secondary stage), and which differs, in its exceptional cases, somewhat from its more short-lived congeners by being followed by sequellæ (to which we give the name of tertiary signs). It is uncertain yet as to whether the virus is a microbe; Lustgarten affirms that there is a special one belonging to this disease, while Dr. Smirnoff, of Kazan, states that he can not find any specialised organisms, as micro-organisms are found quite as often in the normal genital secretions as in syphilis. The division of syphilis into three stages is also embarrassing, as there are no clearly marked lines upon which to state definitely when they end and when they begin. The simplest plan is to call the primary stage, that before the blood is affected; the secondary, during the period of blood infection; and to treat the third as a sequel of the secondary only, and not as a separate stage of its own. The limit of the first is not known, but the secondary is usually taken to terminate when the symmetry of the eruptions cease, and this again depends upon the effect of treatment and the idiosyncrasy of the individual; but for the treatment, says a leading surgeon, syphilis would run its course with the exactitude of variola in all its stages. In the transmission of this disease many very interesting points are raised, while the data upon which our opinions are founded are varied and sometimes amusing. Firstly, there is the question, can the poison be communicated through an unbroken surface? I think a negative answer may be given to this, the great majority of opinions say that it cannot, and when we consider the number of nurses or students who are daily in contact with the disease, surely some authentic instance would soon come before our notice. Ricord also quotes a case to prove the breach of surface is necessary for the absorption of the poison. The time which exists between the reception of the poison and the absorption is very short, probably reception and absorption taking place simultaneously, nor is there anything which will eradicate the disease in the absorption stage. The most absolute cleanliness will not suffice, as the following story of a French medical student will show, having put himself in the way of getting syphilis and immediately after coitus carefully washed himself, but to no avail, for after the usual time of incubation a hard sore developed upon the former seat of a small erosion. Even profuse bleeding from an excoriation so as to almost preclude the entrance of the poison is no safeguard against catching the disease. Thus we may say that the virus of syphilis is inoculable only through an eroded surface, that the time required for its absorption is extremely short, and remembering the delicacy of the epiltulial covering of the genital organs we cannot be surprised at the ease with which the poison is conveyed from one to another. There are three ways in which syphilis can be transmitted: (1) immediate or direct contagion; (2) mediate or indirect contagion; (3) heredity. 1. I will first give a few instances of the immediate or direct contagion, the most common, that of "coitus," requiring no further comment. (*a*) Midwifery opens up a field at once. A medical man or a nurse is in attendance upon a syphilised patient and unacquainted with the fact, having a small scratch, broken hangnail, or excoriation, and being either too lazy or too careless

### The Hospital Advertiser.

### THE STUDENT OF MEDICINE—(*continued*).

do not protect themselves, and thus unheedingly become innoculated with the poison. In the case of nurses, who, perhaps, have not been so well educated up to a sense of their responsibility in isolating themselves from all patients, and refusing to attend confinements until after they are thoroughly well, the disease may be spread right and left; giving it in the first place to the parturient women whom they are nursing, and these in turn passing it on, as an annuity, to their babies and husbands. In these days of nursing lectures and training institutions, one hopes that such a thing could never be, as it would show great ignorance in those calling themselves nurses, and would tend to give the Midwives Registration Bill a stronger hold in the minds of its supporters. To show that these are not imaginary evils, I quote Ricord's case of a nurse who managed to infect over one hundred people with primary sores, owing to her ignorance or dishonesty. (*b*) Again, a wet nurse may be infected in another way, and that is by means of her foster-children, and in this case the primary sore will appear on the nipple, the poison finding an entrance to the system through some slight crack or abrasion, which may, again, have been caused by stomatitis in the infant's mouth. From this source of infection their own babies and husbands, and even, in some instances, the elder children who have been minding the baby have become infected. (*c*) Conversely see how much harm may be done by the introduction of a tainted wet nurse into a healthy household, infecting first the baby, and then the other members of the household. A baby with a sore mouth would do incalculable harm, as they are usually the centre of attraction, not only for that household but for all the female part of their relations. And supposing that an infected nurse were engaged for some institution similar to a foundling hospital immense harm might be done before it was detected. Or perhaps, as might happen in a poorer household, a woman whose baby is healthy may have a neighbour infected, who, hearing the child scream, in a moment of compassion puts it to her own breast, and thus starts the centre of another epidemic. I shall quote a case further on when speaking of vaccino-syphilis. I have laid stress on these points because they are of practical every-day importance, showing how careful we should be in the lying-in chamber, and in the choice of a wet nurse, who should be healthy, of a fresh complexion, with healthy infants, not jaded or worn out. Eradicate any suspicion of secondaries, and look to see that that the infants have no mucous patches or eruptions on the buttocks, for syphilitic children are not always wizened, but where there are local manifestations may grow well and get plump (Mr. Hutchinson).

### APPOINTMENTS.

At the Bristol City Asylum, Frederick Edward Rainsford, B.A., L.R.C.P., has been appointed second assistant medical officer. At the Royal Albert Edward Infirmary and Dispensary, C. Carter Weldon, M.D. Lond., M.R.C.S., has been appointed senior house surgeon. At the Horton Infirmary, Bandbury, E. F. Manwaring, M.R.C.S., L.R.C.P., has been appointed house surgeon. At the St. Pancras Infirmary, Arthur B. Kingsford, L.R.C.P., M.R.C.S., has been appointed junior assistant medical superintendent. At the Central London Ophthalmic Hospital, C. Norman Hamper, M.B., C.M.Edin., has been appointed house surgeon. At the Stanley Hospital, Liverpool, J. Guest Cornall, M.R.C.S., L.R.C.P., B.A. Cantab., has been appointed house surgeon.

### VACANCIES.

The following vacancies are announced this week, the amount of salary in each case being given after the name of the institution:

**Resident Medical Officers.**

Coventry and Warwick Hospital, Assistant to the House Surgeon for six months—Honorarium of £15, and board, &c.
Huddersfield Infirmary, Junior House Surgeon—Salary £40, with board, &c.

**Non-Resident Medical Officers.**

Brighton and Hove Lying-in Institution, Honorary Surgeon-in-ordinary.
Queen's College, Birmingham, Lecturer on Operative Surgery.
Singapore, Straits Settlements, Health Officer—Salary 300 dols. per mensem, and house allowance.
Worcester Amalgamated Friendly Societies' Medical Association, Assistant Medical Officer—Salary £140, with portion of fees, and £20 for cab hire.

### SCHOLARSHIPS AND PRIZES.

**University of Aberdeen—Summer Session, 1891.**

CLASS OF BOTANY (STUDENTS OF FIRST YEAR).

PRIZEMEN.—R. Robertson, A.M., B.D., Aberdeen, 93·2 per cent.; H. Duffus, A.M., New Pitsligo, 88·8; R. G. Henderson, Keith, 88.

### The Hospital Advertiser.

### THE STUDENT OF MEDICINE—(*continued*).

Certificates.—W. B. Davidson, A.M., Beauly, 78 8; A. H. Mackie, A.M., Aberdeen, 77·6; J. Dawson, A.M., Rathen, Lonmay, 70·8; W. Cowie, Ordiquhill, 69·6; W. G. Grant, Salterhill, Elgin, 69·2; R. Lindsay, Tomintoul, 68; J. A. Black, A.M., Aberdeen, 66·4; A. P. Rust, Peterculter, 66; G. A. Reid, Beauly, 65·6; G. B. Scott, Peterculter, 65·2; J. S. Warrack, Stranraer, 60·4; J. A. Mearns, Kinnellar, 60; E. K. Gawn, Whaley Bridge, Stockport, Cheshire, 59·2; J. Duncan, Aberdeen, 58·8.

Students of Second Year.

Prizemen.—A. T. Gage, A.M., Aberdeen, 92·8; P. Macdonald, A.M., Edinburgh, 88; G. Bruce, A.M., Keith Hall, 86·4; W. Craib, Macduff, W. C. Hossack, St. Catherine's, Banff—equal, 86; G. Cowie, Ordiquhill, 84·8.

Certificates.—R. Smith, Friockheim, 77 2; W. M. Keith, Turriff, 75·2; G. Grant, A.M., Grantown, 74·8; W. E. G. Duthie, A.M., Woodside, and J. A. Gordon, Dingwall—equal, 73·6; A. Archibald, A.M., Buckie, 71·2; G. C. Grant, Dufftown; 69·2; P. Mackessack, Kinloss, Forres, 66·4; G. A. Troup, Aberdeen, 61·6.

CLASS OF PRACTICAL BOTANY.

Certificates.—W. Craib, Macduff, 85; G. Cowie, Ordiquhill, 80; A. Mowat, Invergordon, 78; G. M. Duncan, London, 76; J. Dawson, A. M., Rathen, Lonmay, 74; N. J. Sinclair, Old Aberdeen, 63.

NATURAL HISTORY (Junior Division).

Silver Medal.—J. B. Smith, Ardgrain, Ellon.

Bronze Medals.—W. Cartwright, Limefield, Blackburn, and G. A. Reid, Bruriach, Beauly—equal.

First Class Certifcates.—A. Reid, Portgordon; A. P. Rust, Peterculter; and A. Mowat, Invergordon—equal. L. J. Helmrich, Aberdeen; G. C. Grant, Dufftown; R. A. Coles, Warminster, Wilts; and H. R. Gardner, Portsmouth—equal.

Second Class Certificates.—A. J. Hynd, Aberdeen; G. A. Troup, Rhynie; R. Lindsay, Tomintoul; G. B. Scott, Peterculter; G. A. Gibb, Cruden, and J. Duncan, Aberdeen—equal; H. Barraclough, Leeds; J. W. Milne, New Deer, and J. W. Myers, Leeds—equal; W. Cowie, Ordiquhill, and T. D. Cumming, Aberdeen—equal; P. W. Ironside, Stromness, and J. H. Rowe, Hayle, Cornwall—equal; G. Cowie, Ordiquhill; V. Van Langenberg, Colombo; H. Cowan, Inverness, P. J. Henderson, Blairgowrie, and J. A. Mearns, Kinellar—equal; W. Lethbridge, Bedford; A. Falconer, Aberdeen; T. D. Bright, Freetown, Sierra Leone; C. H. Roy, Hepburn-on-Tyne; J. Skinner, Insch.

Senior Division (Third Year Students).

First Class Certificates.—A. Don, Strathdon, and G. Bruce, Inverurie—equal; T. G. Bonner, Aberdeen; R. Cruden, Inverurie.

Second Year Students.

Silver Medal.—A. T. Gage, Aberdeen.
Bronze Medal.—W. C. Hossack, Banff.

First Class Certificates.—P. MacDonald, Edinburgh; T. M'Ross, Aberdeen; J. A. Black, Aberdeen; and J. S. Warrack, Stranraer—equal; J. L. Salmond, Aberdeen; J. A. Gordon, Dingwall; G. Mitchell, Keith; and R. Smith, Friockheim—equal; J. G. Jones, Elgin.

Second Class Certificates.—W. M. Keith, Turriff; J. M'Andrew, Fife-Keith; H. Duffus, New Pitsligo; J. S. Laing, Aberdeen; J. MacPherson, Stalybridge; and A. Sharp, Cultercullen—equal; G. Reid, Buckie; G. Thomson, Fettercairn; R. F. Campbell, Wark-on-Tyne, and A. D. Kelly, Aberdeen—equal; Ernest Barnes, Manchester; J. Dawson, Lonmay, and J. Halley, Aberdeen—equal; R. G. Brown, Aberdeen, and D. Ross, Aberdeen—equal.

PRACTICAL NATURAL HISTORY.

Medallist.—A. Don, Strathdon.
First Class Certificate.—P. Macdonald, Edinburgh.
Second Class Certificates.—A. Low; R. Robertson, Aberdeen; W. Craib, N. J. Sinclair, Aberdeen, and R. A. Coles, Warminster—equal.

PRACTICAL CHEMISTRY.

Silver Medal.—E. Oliphant, Cullen, 95.
Bronze Medals.—Andrew D. Kelly, Aberdeen; Arthur Hugh Lister, B.A., Leytonstone, Essex; and William Thomson, Cullen—equal, 88.
First Class Certificates.—Ernest Barnes, Manchester, and George Reid, Buckie—equal, 78; Thomas M'Kay Ross, Aberdeen, 77; William M. Keith, Turriff, and George Thomson, Fettercairn—equal, 75.
Second Class Certificates.—David Ross, Aberdeen, 72; Robert Cruden, M.A., Inverurie, 71; James Dawson, M.A., Lonmay; John L. Dickie, Aberdeen; Alexander Reid, Portgordon; Robert Smith, Friockheim; and Joseph H. Rowe, Cornwall—equal, 70; Robert J. Brown, Aberdeen; Robert F. Campbell, Wark-on-Tyne; Alexander, Don, M.A., Strathdon; W. E. G. Duthie, M.A., Woodside, and W. Philip, M.A., Inverurie—equal 66; W. Craib, Macduff, and G. M. Duncan, London—equal 65; L. Adam, Banchory; T. I. Bonner, M.A., Aberdeen, and G. A. Troup, Rhynie—equal, 64; A. P. Rust, Peterculter, 61; A. Archibald, M.A., Buckie, G. Grant, M.A., Grantown, and J. S. Marr, Drumoak—equal, 60.

### PASS LISTS.

### University of London.

INTERMEDIATE EXAMINATION IN MEDICINE: July, 1891.

Examination for Honours.

Anatomy.—*First Class*: J. Stephenson, B.Sc. (exhibition and gold medal), Owens College; J. S. Collier, B.Sc. (gold medal), St. Mary's Hospital; J. S. Sloane, B.Sc., St. Bartholomew's Hospital. *Second Class*: H. B. Dickinson, University College, Liverpool; A. Byers, B.Sc., Owens College; S. W. F. Richardson, St. Thomas's Hospital. *Third*

### THE STUDENT OF MEDICINE—(*continued*).

*Class*: T. T. Jeans, Owens College and Manchester Royal Infirmary; T. B. Stedman, University College; A. E. Russell, St. Thomas's Hospital; E. H. Houfton, The Yorkshire College.

Physiology and Histology.—*First Class*: J. Le Mare Bunch, B.Sc. (exhibition and gold medal), University College; J. Stephenson (gold medal), Owens College; ‡W. d'Este Emery, Queen's College, Birmingham; ‡J. S. Sloane, St. Bartholomew's Hospital. *Second Class*: H. B. Dickinson, University College, Liverpool; R. A. Young, Middlesex Hospital; J. S. Collier, St. Mary's Hospital; ‡A. Byers, Owens College; ‡A. H. Leete, Guy's Hospital. *Third Class*: F. J. Fielder, King's College; S. W. F. Richardson, St. Thomas's Hospital; ‡W. R. Bryett, B.A., King's College; ‡A. M. Collcutt, Cambridge University; A. E. Wilson, St. Mary's Hospital.

Organic Chemistry.—*First Class*: J. A. Howard (exhibition and gold medal), Guy's Hospital; * Elizabeth J. Moffett, B Sc., London School of Medicine for Women; H. Sinigar, Queen's College, Birmingham. *Second Class*: J. S. Collier, St. Mary's Hospital.

Materia Medica and Pharmaceutical Chemistry.—*First Class*: G. H. Cowen (exhibition and gold medal), London Hospital; W. d'Este Emery, Queen's College, Birmingham. *Second Class*: R. A. Young, Middlesex Hospital; L E V. Every-Clayton, Guy's Hospital. *Third Class*: J. A. Howard, Guy's Hospital; J. S. Collier, St. Mary's Hospital; J. S. Sloane, St. Bartholomew's Hospital; K. Rogers, St. Bartholomew's Hospital; T. B. Steadman, University College.

*Obtained the number of marks qualifying for the Exhibition.
†Obtained the number of marks qualifying for a Gold Medal.
‡Denotes equality of merit.

### INTERMEDIATE SCIENCE AND PRELIMINARY SCIENTIFIC (M.B.) CONJOINTLY.

Inorganic Chemistry.—*First Class*: H. D. Fuge, Int.Sc. (disqualified by age for the exhibition), Pharmaceutical Society, Birkbeck Institute, and private study; H. Grime, Int.Sc., Royal College of Science and University Tutorial College; C. A. Silberrad, Int.Sc., St. Peter's College, Cambridge. *Second Class*: C. A. White, Int.Sc., Mason College and private study; J. A. Jones, Int.Sc., private study; M. Ware, Int.Sc., Royal College of Science; *R. A. Lyster, Prel.Sc., Mason College; *D. N. Nabarro, Prel.Sc., Owen's School, Islington, and University College. *Third Class*: F. Cartlidge, Int.Sc., Westminster Training College and Wedgwood Institute; S. Wood, Int.Sc., Royal College of Science and private study; *H. C. Hickinbotham, Prel.Sc., King Edward High School and Mason College, Birmingham; *H. Boulton, Prel.Sc., Epsom College; *W. H. Willcox, Prel.Sc., University College and private study; E. J. Eastes, Int.Sc., private study and Birkbeck Institute; J. W. Cobb, Int.Sc., The Yorkshire College; *W. S. Patch, Prel.Sc., University College and private study.

Experimental Physics.—*First Class*: A. E. Briscoe, Int.Sc. (disqualified by age for the Neil Ar ott exhibition and medal), Royal College of Science and University College, Nottingham. *Second Class*: H. C. Haycraft, Inst.Sc., City Guilds Institution, University Tutorial College and Birkbeck; S. Wood, Int.Sc., Royal College of Science and private study. *Third Class*: C. A. Silberrad, Int.Sc., St. Peter's College, Cambridge; W. G. Burbidge, Int.Sc., Royal College of Science and private study; * A. J. Rodocanachi, Pr.Sc., University College and private tuition; J. D. Thompson, Int.Sc., Owens College; J. W. Cobb, Int.Sc., The Yorkshire College; F. W. Le Tall, Int.Sc., City Guilds Institute and private study; J. J. Stewart, Int.Sc., University College, London, and Emmanuel College, Cambridge; W. R. Jamieson, Int.Sc., University College, Bristol; * E. M. Corner, Prel.Sc., Epsom College.

Botany.—*Second Class*: J. A. Audley, Int.Sc., private study; H. D. Fuge, Int.Sc., Pharmaceutical Society, Birkbeck Institute, and private study; V. H. Blackman, Int.Sc., St. Bartholomew's Hospital. *Third Class*: E. B. Sherlock, Int.Sc., Birkbeck Institution and private study; C. Butcher, Int.Sc., University College; P. Turner, Int.Sc., King's College.

Zoology.—*Second Class*: J. C. Griffiths, Int.Sc., Mason College and private study; F. A. Smith, Int.Sc., St. Bartholomew's Hospital; A. R. J. Douglas, Int.Sc., St. Bartholomew's Hospital. *Third Class*: J. Hussey, Prel.Sc., St. Bartholomew's Hospital.

*Obtained the number of marks qualifying for the exhibition or prize.

† These candidates have also passed in the Mathematics of the Intermediate Examination in Science, and have thus become admissible to the B.Sc. Examination.

‡ Denotes equality of merit.

### Indian Medical Service.

The following is the list of Surgeons on probation who were successful at the recent London and Netley examinations. The prizes are awarded as in the British Medical Service.

| | Marks. | | Marks. |
|---|---|---|---|
| *Barton-Brown, F. H. | 6292 | Wood, H. S. | 5465 |
| Deare, B. H. | 5880 | Irvine, T. W. | 5413 |
| Oldham, B. C. | 5820 | Entrican, J. | 5395 |
| Bird, R. | 5775 | Pridmore, W. G. | 5350 |
| †Smith, S. B. | 5717 | Donovan, C. | 5235 |
| Henvey, W. | 5598 | Penny, J. | 5175 |
| Lumsden, J. S. S. | 5570 | Brown, A. T. | 5160 |
| ‡Frost, G. H. | 5565 | Graves, D. H. McD. | 5120 |
| Wilkinson, E. | 5535 | Dallas, S. A. C. | 5085 |
| Ewens, G. F. W. | 5513 | Palk, C. H. L. | 4905 |
| Duer, C. | 5480 | | |

*Gained the Prize in Pathology presented by Sir Wm. Aitken, F.R.S.

†Gained the Parkes Memorial Bronze Medal in Hygiene.

‡Gained the Martin Memorial Gold Medal and the Prize in Clinical Medicine presented by Surgeon-General W. C. Maclean, C.B.

